# High-Extraction-Rate Ta_2_O_5_-Core/SiO_2_-Clad Photonic Waveguides on Silicon Fabricated by Photolithography-Assisted Chemo-Mechanical Etching (PLACE)

**DOI:** 10.3390/nano14171466

**Published:** 2024-09-09

**Authors:** Jian Liu, Youting Liang, Lang Gao, Chao Sun, Jianglin Guan, Zhe Wang, Zhaoxiang Liu, Zhiwei Fang, Min Wang, Haisu Zhang, Ya Cheng

**Affiliations:** 1 State Key Laboratory of Precision Spectroscopy, East China Normal University, Shanghai 200062, China; 52210920027@stu.ecnu.edu.cn (J.L.); sunchao_3706@stu.ecnu.edu.cn (C.S.); 51200920051@stu.ecnu.edu.cn (J.G.); 2 The Extreme Optoelectromechanics Laboratory (XXL), School of Physics and Electronic Science, East China Normal University, Shanghai 200241, China; ytliang@phy.ecnu.edu.cn (Y.L.); zwang@phy.ecnu.edu.cn (Z.W.); zxliu@phy.ecnu.edu.cn (Z.L.); zwfang@phy.ecnu.edu.cn (Z.F.); mwang@phy.ecnu.edu.cn (M.W.); 3 State Key Laboratory of High Field Laser Physics and CAS Center for Excellence in Ultra-Intense Laser Science, Shanghai Institute of Optics and Fine Mechanics (SIOM), Chinese Academy of Sciences (CAS), Shanghai 201800, China; langgao@siom.ac.cn; 4 Collaborative Innovation Center of Extreme Optics, Shanxi University, Taiyuan 030006, China; 5 Collaborative Innovation Center of Light Manipulations and Applications, Shandong Normal University, Jinan 250358, China; 6 Hefei National Laboratory, Hefei 230088, China; 7 Shanghai Research Center for Quantum Sciences, Shanghai 201315, China

**Keywords:** tantalum pentoxide, femtosecond laser direct writing, high extraction rate, low scattering loss, microring resonator

## Abstract

We demonstrate high-extraction-rate Ta_2_O_5_-core/SiO_2_-clad photonic waveguides on silicon fabricated by the photolithography-assisted chemo-mechanical etching technique. Low-confinement waveguides of larger than 70% coupling efficiency with optical fibers and medium propagation loss around 1 dB/cm are investigated in the experiment. Monolithic microring resonators based on Ta_2_O_5_ waveguides have shown the quality factors to be above 10^5^ near 1550 nm. The demonstrated Ta_2_O_5_ waveguides and their fabrication method hold great promise in various cost-effective applications, such as optical interconnecting and switching.

## 1. Introduction

Tantalum pentoxide (Ta_2_O_5_) is a well-known dielectric material used in high-quality mirror coatings for optical experiments requiring high laser power [1]. The CMOS compatibility of such material also enables a large-scale photonic integration of optical waveguides and resonators, which are further profited by the high refractive index (~2.05 at 1550 nm), large bandgap (~4 eV), low optical loss, and broad transmission range (300 nm–8 μm) of Ta_2_O_5_ [2,3]. In addition, compared with the prevalent silica and silicon nitride (Si_3_N_4_) photonic integration platform, the nonlinear refractive index of Ta_2_O_5_ has been found to be an order of magnitude and three times higher than these two materials, respectively [4,5,6]. Such an advantage facilitates various nonlinear applications, such as supercontinuum generation and optical switching, to be realized in reduced interaction lengths and thus a more compact footprint with high scalability [7,8,9,10,11,12,13,14,15,16].

Waveguide fabrication in Ta_2_O_5_ has progressed rapidly in the last decade. Due to the low film stress of Ta_2_O_5_ compared with Si_3_N_4_, fabricating either low-mode-confinement waveguides with ~100 nm thickness or high-mode-confinement waveguides with 500–800 nm thickness is quite flexible [3,11,12,13,14,15,16]. Both waveguide configurations have been employed to realize propagation losses as low as 3 dB/m, which are determined by the residual interface roughness resulting from the dry etching process [3]. In this work, by using the photolithography-assisted chemo-mechanical etching (PLACE) technique [17,18], Ta_2_O_5_-core/S_i_O_2_-clad waveguides on a silicon substrate are fabricated with a very low surface roughness of ~1 nm. This kind of Ta_2_O_5_ waveguide supports high coupling efficiency with the ultrahigh-NA optical fiber and the retrieved coupling loss between the waveguide, and the fiber is 1.3 dB at 1550 nm by the cut-back method, while the waveguide propagation loss is deduced to be 1 dB/cm. The high waveguide propagation loss is discussed to be induced by the inherent absorption loss in the deposited Ta_2_O_5_ thin film and the oxide cladding layer. Microring resonators based on Ta_2_O_5_ waveguides are also fabricated by the PLACE technique, showing quality factors above 10^5^ at 1550 nm. Further reduction in material losses through optimized film deposition process can enable large-scale applications of the Ta_2_O_5_ waveguides fabricated by the high-throughput PLACE technique.

## 2. Experimental Details

**Figure 1:**
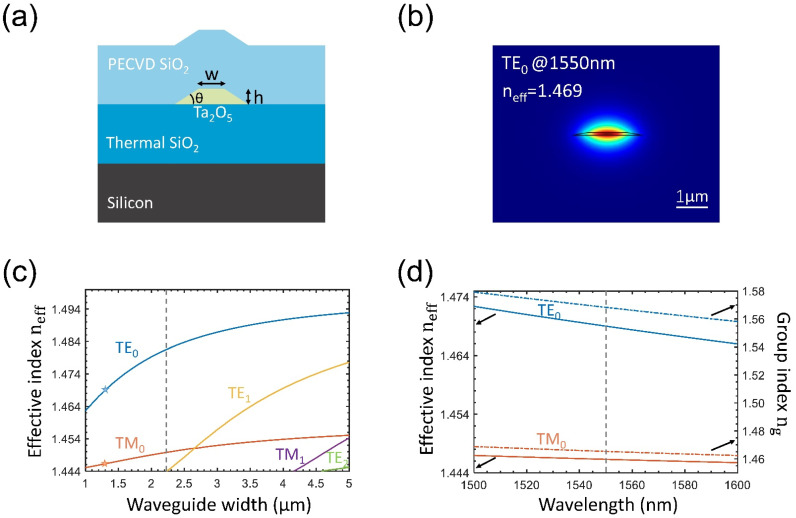
Design and property of Ta_2_O_5_ waveguide: (**a**) a cross-section schematic of the Ta_2_O_5_ waveguide, (**b**) the simulated fundamental TE-mode profile of the Ta_2_O_5_ waveguide at 1550 nm, (**c**) the effective refractive index vs waveguide width for different modes, (**d**) the effective index and group index vs wavelength for the fundamental modes.

Figure 1a shows a schematic of the Ta_2_O_5_-core/S_i_O_2_-clad waveguide. A Ta_2_O_5_ thin film is first deposited on the thermally oxidized silicon wafer, and then the Ta_2_O_5_ layer is patterned into the waveguide core, with the ensuing oxide over-cladding deposited by the plasma-enhanced chemical vapor deposition process (PECVD). Similar waveguide configurations have been validated in the low-loss Si_3_N_4_ platform [19]. The simulated fundamental transverse electric (TE) mode profile of the Ta_2_O_5_-core/S_i_O_2_-clad waveguide is shown in Figure 1b. The simulation is completed with the finite-element method (FEM), using the refractive index distribution in the waveguide region (n_clad_ = 1.444 for SiO_2_ and n_core_ = 2.058 for Ta_2_O_5_ at λ = 1550 nm). The width and height of the Ta_2_O_5_ core are selected to be w = 1.3 μm and h = 96 nm in the simulation, in accordance with the profile of the fabricated waveguides tested later. In addition, the Ta_2_O_5_-core sidewall angle is set to θ = 10° in accordance with the fabricated sample. It can be seen from Figure 1b that most of the guided optical power is distributed in the SiO_2_-clad, giving the waveguide an effective index of $n_{eff}$ = 1.469. The modal propagation properties of the Ta_2_O_5_ waveguide are further simulated at different widths and wavelengths while keeping the core height and sidewall angle constant (h = 96 nm and θ = 10°). The results are shown in Figure 1c,d. The single-mode propagation condition (ignoring polarization dependence) is clearly illustrated in Figure 1c, where only the fundamental modes (TE_0_ and TM_0_) are allowed to propagate when the waveguide width is less than 2.2 μm. The waveguide dispersion curves for the TE_0_ and TM_0_ modes at the waveguide width of 1.3 μm (the star points in Figure 1c) are further shown in Figure 1d, and the corresponding group index dispersion curves are also plotted, giving the group index of $n_{g}$ = 1.568 and $n_{g}$ = 1.448 at 1550 nm for the TE_0_ and TM_0_ modes, respectively.

**Figure 2:**
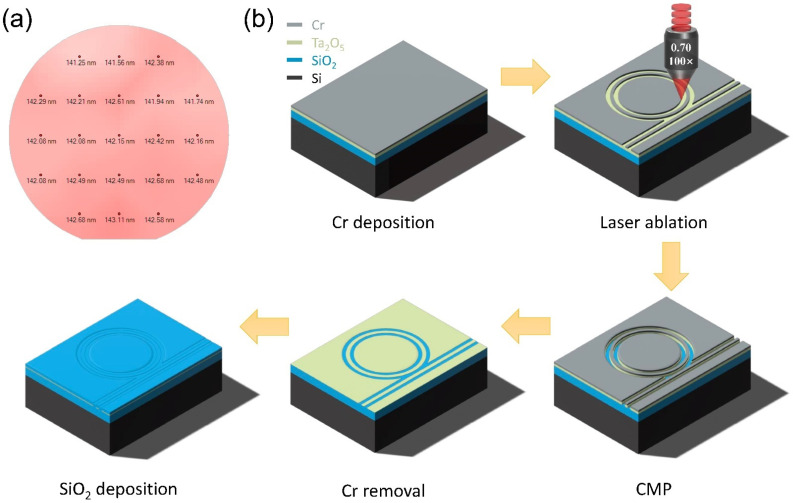
Fabrication processes of the Ta_2_O_5_ waveguide: (**a**) the thickness profile of the deposited Ta_2_O_5_ film, (**b**) the process flow for the Ta_2_O_5_ waveguide fabrication.

The Ta_2_O_5_ thin film is deposited by electron-beam evaporation (EBE) method on the thermally oxidized silicon wafer [20]. The silicon wafer has a diameter of 4 inches and a thickness of 450 μm, with 10-μm-thick oxide layers at both the top and bottom faces. The EBE process is conducted at a temperature of 280 °C and an oxygen-filled vacuum pressure of 2.5 × 10^−2^ Pa. The evaporation rate is 3 A/s with an ion beam flux of 30 mA. The surface profile of the deposited Ta_2_O_5_ film is examined by the profilometer, and the result is shown in Figure 2a. An average film thickness of 141 nm with ±1 nm fluctuation is achieved. The Ta_2_O_5_ waveguide is fabricated with the process flow shown in Figure 2b. A thin layer of chromium is first deposited on the Ta_2_O_5_ film by magnetron-sputtering at room temperature. The deposition rate is 2 A/s in the argon-filled environment of 2.1 × 10^−1^ Pa. The thickness of the deposited chromium film is 250 nm. Using femtosecond laser ablation, the chromium thin film is prepared into the desired mask pattern. The employed laser parameters are a central wavelength of 1030 nm, a single pulse width (full width at half maximum, FWHM) of 190 fs, and an average laser power of 0.08 mW, with a repetition rate of 250 kHz. The laser beam is tightly focused onto the sample by a microscope objective (NA = 0.7, 100×, M Plan Apo NIR, Mitutoyo Corporation, Kawasaki, Kanagawa, Japan) at ambient conditions (i.e., room temperature, dry air), and the resolution of the laser ablation is about 200 nm [17]. The sample is mounted on an air-bearing motorized stage (Aerotech, Inc., Pittsburgh, PA, USA), with a translation resolution of 100 nm and moving with a speed of 1 mm/s during the femtosecond laser ablation. 

The laser-ablated chromium mask pattern is transferred onto the Ta_2_O_5_ layer underneath by chemo-mechanical polishing (CMP). The CMP process is conducted using a wafer polishing machine (NUIPOL802, Kejing, Inc., Hefei, China) with a piece of velvet polishing cloth. An amorphous colloidal silica suspension with a particle diameter of 60 nm is used as the polishing slurry. The polishing time is about 7 min. Then, the residual chromium mask is removed by immersion in a standard Cr-etching solution for 10 min. An additional CMP is conducted to further smooth the edge of the fabricated structure, which can attain a high surface quality comparable with the surface tension limited roughness. The height of the Ta_2_O_5_ structure after the second CMP is about 100 nm. Afterwards, an oxide layer of 3.5 μm thickness is deposited on top of the fabricated structure by PECVD. The PECVD process is carried out by the Oxford PlasmaPro-100 PECVD (Oxford Instruments, Abingdon, Oxon, UK) equipment at a temperature of 300 °C, a deposition rate of 58 nm/min, and an RF power of 20 W. The environment pressure is 1000 mtorr, with an SiH_4_ flux of 710 sccm and a N_2_O flux of 170 sccm. 

## 3. Results and Discussion

**Figure 3:**
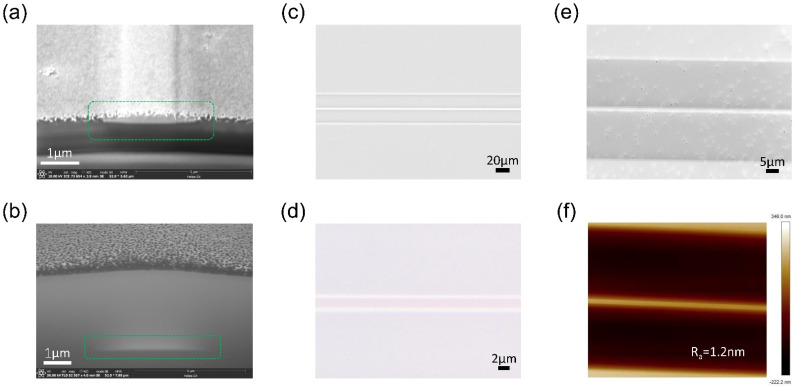
Microscopy characterization of the Ta_2_O_5_ waveguide: (**a**) SEM image of the Ta_2_O_5_ waveguide before oxide cladding deposition, (**b**) SEM image of the Ta_2_O_5_ waveguide after oxide cladding deposition, (**c**,**d**) top-view optical microscope images of the Ta_2_O_5_ waveguide, (**e**) top-view SEM image of the Ta_2_O_5_ waveguide, (**f**) top-view AFM image of the Ta_2_O_5_ waveguide. The region in the green dashed boxes is the Ta_2_O_5_ core layer.

Scanning electron microscopy (SEM) is first used to characterize the fabricated Ta_2_O_5_ waveguide. The waveguide cross-section images are shown in Figure 3a,b. The Ta_2_O_5_-core profile before the deposition of oxide cladding is shown in Figure 3a, featuring a slant sidewall with small angles (~10°) typical of the PLACE technique. The full profile of the Ta_2_O_5_-core/S_i_O_2_-clad waveguide is shown in Figure 3b, where the small Ta_2_O_5_ core is labeled in the green dashed box for clarity. The optical micrographs of the Ta_2_O_5_ waveguide are further shown in Figure 3c,d, and the SEM image of the Ta_2_O_5_ waveguide is shown in Figure 3e. The waveguide surface profile is further measured by atomic force microscopy (AFM), with the result shown in Figure 3f. The residual surface roughness is deduced to be R_a_ = 1.2 nm from the AFM measurement. Such a level of surface roughness will induce very little scattering loss (<0.1 dB/cm) for waveguide propagation.

**Figure 4:**
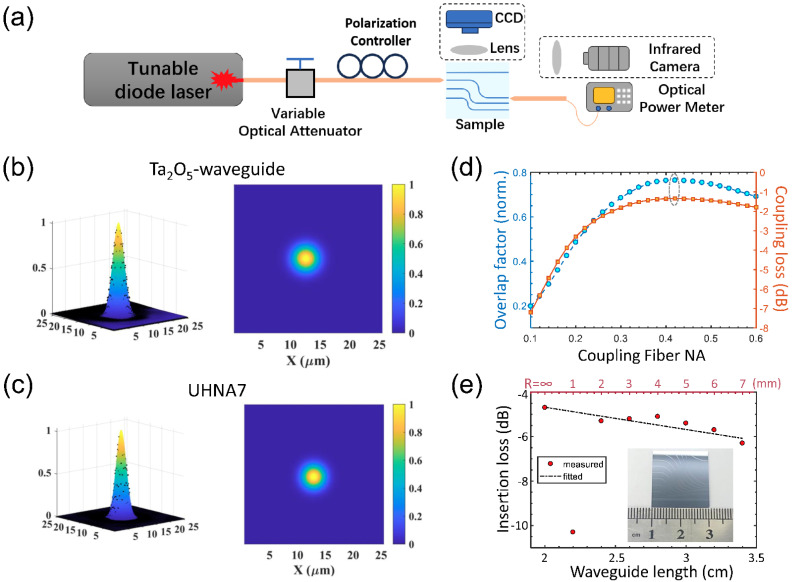
Guided mode and propagation loss: (**a**) the experimental setup for waveguide mode and loss measurement, (**b**) the output mode profile of the Ta_2_O_5_ waveguide, (**c**) the output mode profile of the UHNA7 fiber, (**d**) the calculated mode overlap factor and coupling loss between the Ta_2_O_5_ waveguide and the UHNA7 fiber, (**e**) the measured insertion losses of the Ta_2_O_5_ waveguide of variable lengths and bending radii.

The output mode profile of the Ta_2_O_5_ waveguide is measured by the microscope imaging system equipped with an infrared charge-coupled device (InGaAs camera, HAMAMATSU, Inc., Hamamatsu City, Shizuoka, Japan), as shown in Figure 4a. The input light is coupled into the waveguide using an ultrahigh-NA fiber (UHNA7) with NA = 0.41 and a mode field diameter (MFD) of 3.2 μm at 1550 nm. An in-line fiber-based polarization controller is used to adjust the input polarization for the TE-mode excitation in the Ta_2_O_5_ waveguide. The output light from the waveguide is collected by the microscope objective (NA = 0.2, 10×) and imaged into the infrared camera. The measured mode profile at 1550 nm is depicted in Figure 4b, where the 2D and 3D color views are given in the right side and left side, respectively. For comparison, the output mode profile from the UHNA7 fiber is also measured by the same imaging system and is shown in Figure 4c. Similar mode profiles from the Ta_2_O_5_ waveguide and UHNA7 fiber can be noticed, indicating the high coupling efficiency between the two components.

The mode field overlaps between the Ta_2_O_5_ waveguide and the optical fibers of variable NAs are calculated using the overlap analysis where the fiber modes are approximated by vectorial Gaussian beams in the calculation [21]. The results are shown in Figure 4d. The largest overlap factor is 75% for the fiber, with an NA = 0.42, which is quite close to the UHNA7 fiber used in the experiment. The coupling losses between the waveguide and the fiber, considering the mode overlap and the index mismatch (Fresnel reflection), are also calculated and shown in Figure 4d. The minimum coupling loss is about 1.3 dB for the UHNA7 fiber, denoted by the dashed elliptical circle in Figure 4d.

The waveguide propagation losses are characterized by the cut-back method, using the same experiment setup shown in Figure 4a. A series of Ta_2_O_5_ waveguides with identical profiles are fabricated. To reduce the required footprint, waveguides longer than 2 cm are designed to have curved sections. The bending radii for the waveguide lengths of 2.2 cm, 2.4 cm, 2.6 cm, 2.8 cm, 3.0 cm, 3.2 cm, and 3.4 cm are 1 mm, 2 mm, 3 mm, 4 mm, 5 mm, 6 mm, and 7 mm, respectively. The measured fiber-to-fiber insertion losses of the Ta_2_O_5_ waveguides are shown in Figure 4e, and the inset shows the digital picture of the sample under test. A linear fit to the measured data, excluding the second point, gives a coupling loss of 1.3 dB per facet and a propagation loss of 1 dB/cm. The large deviation of the second data point should come from the small bend radius used (1 mm), while the consistence of the unit propagation losses of curved waveguides with straight waveguides implies the bending loss is very little when the bending radius is greater than 2 mm.

The measured relatively high propagation loss of 1 dB/cm for the Ta_2_O_5_-core/SiO_2_-clad waveguides should mainly come from the inferior quality of the film deposition process since the scattering loss incurred by the interface roughness is greatly suppressed through the PLACE fabrication technique. Ta_2_O_5_ film deposition by electron-beam evaporation can reduce the intrinsic stress, though the residual metal contaminants could induce absorption loss. In addition, the OH absorption induced by the oxide cladding deposition through PECVD has long been a problem for photonic devices working around the telecom C-band. The employed low-mode-confinement Ta_2_O_5_ waveguide is very susceptible to the absorption loss in the oxide cladding. High temperature annealing can remedy such a problem, though the annealed temperature is unfeasible with the employed Ta_2_O_5_ waveguide configuration. Further optimizations on the film deposition processes are required to reduce the attainable propagation loss fabricated by the PLACE technique. 

**Figure 5:**
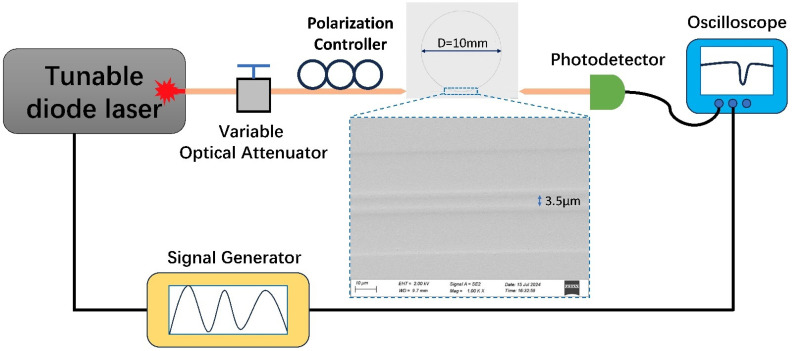
Microresonator characterization setup. The microscope image of the fabricated microring resonator is shown in the middle. Red lines denote a fiber connection and black lines represent an electrical connection.

Monolithic microring resonators coupled with bus waveguides are also fabricated, employing the Ta_2_O_5_-core/S_i_O_2_-clad waveguide and the PLACE technique. Such microresonators have great use in high-power and high thermal-load applications [22]. To minimize the bending loss, the diameter of the microring is set to 10 mm. The optical microscope image of the microring is shown in the middle of Figure 5, and the enlarged SEM image for the coupling region between the microring waveguide and the bus waveguide is shown as well. The coupling gap is 3.5 μm. The transmission spectrum of the microring is measured using a tunable C-band external cavity diode laser (Toptica). The input and output lights are coupled through the UHNA-7 fibers into the bus waveguides. The output lights are sent to a photodetector connected with an oscilloscope to record the transmission spectrum during wavelength scanning. To characterize the overall mode structure, a broadband spectrum is first measured by coarse scanning the input wavelength through mechanically changing the external cavity length of the tunable laser. Then, a triangular wave signal, generated by a signal generator, is applied to the high-resolution piezo-electric movement accessory of the laser for fine wavelength tuning to measure the resonance profile of each mode. 

**Figure 6:**
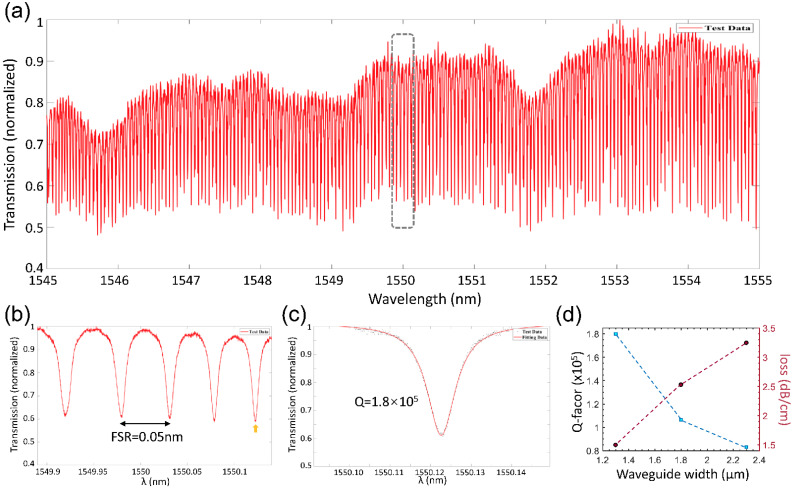
Microresonator Q-factor characterization. (**a**) The full transmission spectrum of the microresonator from 1545 nm to 1555 nm. (**b**) The enlarged part of the transmission spectrum showing the FSR of 0.05 nm. (**c**) The Lorentz fitting of the resonance profile around 1550.12 nm. (**d**) The Q-factors and corresponding propagation losses retrieved from microring resonators of different waveguide widths.

A dense spectrum of optical resonance is measured from 1545 nm to 1555 nm, and the results are shown in Figure 6a. An enlarged spectrum, around 1550 nm, is further shown in Figure 6b, where the free spectral range (FSR) of the microring resonator is found to be 0.05 nm from the spacing between adjacent resonances. The expected FSR can be calculated, using the equation $∆\lambda =\lambda^{2}/\left(\pi D\times n_{g}\right)$ (D = 10 mm is the diameter of the microring resonator, and $n_{g}$ = 1.568 is the group index of the waveguide obtained from the simulation), to be 0.048 nm, which is close to the retrieved FSR in the experiment. The transmission profile of a single resonance curve around 1550.12 nm is fitted by the Lorentz function shown in Figure 6c, from which the quality factor is obtained to be Q = 1.8 × 10^5^. The microring resonator with different waveguide widths is also fabricated and characterized. From the measured Q-factors, the waveguide propagation losses are extracted by the equation $\alpha =2\pi n_{g}/\left(\lambda \times Q\right)$ and shown in Figure 6d. The Q-factors range from 0.8 × 10^5^ to 1.8 × 10^5^, and the corresponding propagation losses are from 3.2 dB/cm to 1.5 dB/cm. The higher propagation losses retrieved from the microring resonator Q-measurement could come from the unsymmetrical coupling region due to the anisotropic chemical polishing process, which could be improved by further optimization of the coupling region. 

## 4. Conclusions

In conclusion, we demonstrated the low-mode-confinement Ta_2_O_5_-core/S_i_O_2_-clad waveguides of high coupling efficiency with an ultrahigh-NA optical fiber, fabricated by the photolithography-assisted chemo-mechanical etching technique. Smooth waveguides are revealed in the experiment, and the measured waveguide propagation loss of 1 dB/cm is dominated by the inherent material absorption. Future improvements by adopting an optimized film deposition process could reduce the waveguide loss to very low values. The demonstrated waveguides hold great promise in various nonlinear optical applications requiring high power and a high extraction rate.

## Data Availability

Data underlying the results presented in this paper are not publicly available at this time but may be obtained from the authors upon reasonable request.
